# Gliotransmitter Release from Astrocytes: Functional, Developmental, and Pathological Implications in the Brain

**DOI:** 10.3389/fnins.2015.00499

**Published:** 2016-01-12

**Authors:** Kazuki Harada, Taichi Kamiya, Takashi Tsuboi

**Affiliations:** Department of Life Sciences, Graduate School of Arts and Sciences, The University of TokyoTokyo, Japan

**Keywords:** astrocytes, exocytosis, glial cell, gliotransmitter, neurodevelopmental disorders, optical imaging, synaptic activity

## Abstract

Astrocytes comprise a large population of cells in the brain and are important partners to neighboring neurons, vascular cells, and other glial cells. Astrocytes not only form a scaffold for other cells, but also extend foot processes around the capillaries to maintain the blood–brain barrier. Thus, environmental chemicals that exist in the blood stream could have potentially harmful effects on the physiological function of astrocytes. Although astrocytes are not electrically excitable, they have been shown to function as active participants in the development of neural circuits and synaptic activity. Astrocytes respond to neurotransmitters and contribute to synaptic information processing by releasing chemical transmitters called “gliotransmitters.” State-of-the-art optical imaging techniques enable us to clarify how neurotransmitters elicit the release of various gliotransmitters, including glutamate, D-serine, and ATP. Moreover, recent studies have demonstrated that the disruption of gliotransmission results in neuronal dysfunction and abnormal behaviors in animal models. In this review, we focus on the latest technical approaches to clarify the molecular mechanisms of gliotransmitter exocytosis, and discuss the possibility that exposure to environmental chemicals could alter gliotransmission and cause neurodevelopmental disorders.

## Introduction

**Figure 1:**
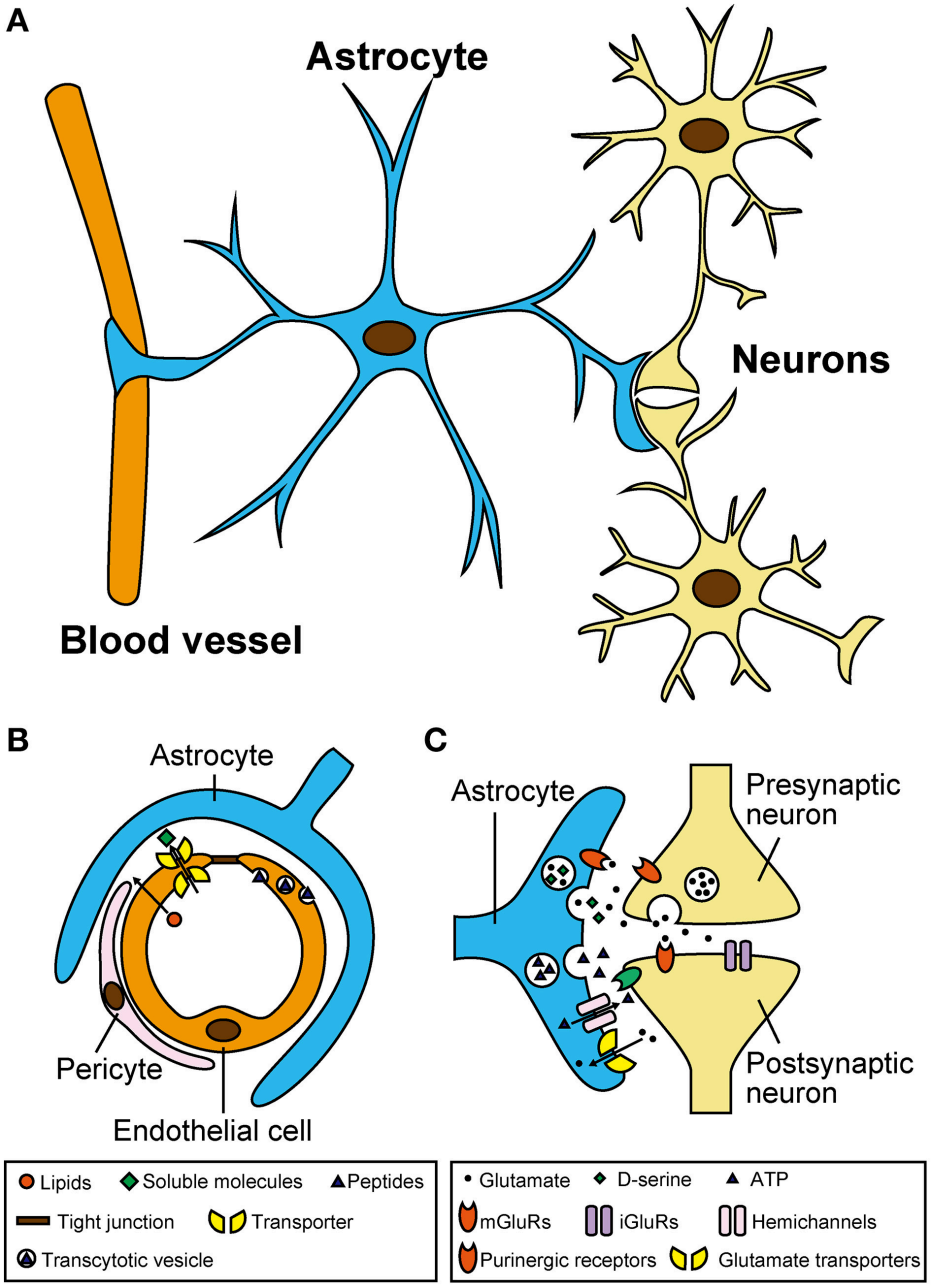
**Astrocytes have close morphological and functional associations with microvasculature and neurons**. **(A)** Location of astrocytes around blood vessels and neurons in the central nervous system. Note that single astrocytes make contact with a large number of blood vessels and neurons through their numerous processes. **(B)** Schematic diagram showing the blood–brain barrier and its functions in selecting and transporting various molecules from the blood stream. Although, vascular endothelial cells form robust tight junctions that prevent infiltration of most soluble molecules, hydrophobic lipids can penetrate across the plasma membrane. In addition, certain soluble molecules such as glucose are actively transported across the endothelial cells via their specific transporters, and some peptides are taken up by selective vesicular transcytosis. **(C)** Schematic diagram showing the tripartite synapse and complex signaling interactions mediated by neurotransmitters and gliotransmitters. Neurotransmitters released from presynaptic terminals such as glutamate act not only on postsynapses but also on astrocytes. Activated astrocytes release gliotransmitters including glutamate, D-serine, and ATP, via vesicular exocytosis (and also possibly via hemichannels for ATP). Released gliotransmitters bind to presynaptic and postsynaptic receptors to regulate synaptic transmission. Astrocytes also take part in clearance of extracellular glutamate via glutamate transporters.

Astrocytes are the most abundant glial cells in the central nervous system (CNS) of mammals (Ventura and Harris, 1999). Based on electron microscopic analyses, astrocytes are located near to neurons and blood vessels (Figure 1A). Regarding vasculature, capillary endothelial cells are surrounded by pericytes and basal lamina, and astrocytes tightly wrap these microvascular structures (Abbott et al., 2006). Together with pericytes, astrocytes are an essential component of the blood–brain barrier (BBB), which selects and transports molecules from the bloodstream, and allows for the transfer of nutrients to neurons (Figure 1B). Regarding their relationship with neurons, astrocytic foot processes make close contact with pre- and post-synaptic areas, forming structures called “tripartite synapses” (Araque et al., 1999; Halassa et al., 2007). Indeed, in the hippocampus, 57% of synapses are associated with astrocytes (Ventura and Harris, 1999), suggesting that astrocytes might contribute to neural information processing in the CNS.

Despite their morphological characteristics described above, astrocytes have long been considered as mere metabolic supporters that nurture adjacent neurons (Halassa et al., 2007; Wang and Bordey, 2008; Calì et al., 2009). However, recent electrophysiology and optical imaging analyses have provided strong evidence that astrocytes respond to neurotransmitters and release chemical transmitters called “gliotransmitters” (Li et al., 2013). Gliotransmitters, including glutamate, D-serine, and ATP, bind to their respective receptors on neurons to modulate their firing frequency and/or synaptic transmission (Figure 1C; Halassa et al., 2007; Koizumi, 2010). In fact, the dysfunction of gliotransmitter release-related proteins (e.g., vesicular transporters and vesicle-associated membrane proteins) in astrocytes can cause serious brain disorders and abnormal behaviors (Rossi et al., 2011; Verkhratsky et al., 2014). At the same time, traumatic injury, stroke, or infection-induced astrogliosis (also known as reactive astrocytes). These reactive astrocytes produce and release neurotoxic levels of glutamate (Rossi et al., 2011; Verkhratsky et al., 2014). Astrocytes also contribute to proper development of the BBB by aligning endothelial cells and pericytes, transporting molecules selected from the bloodstream to neurons (Abbott et al., 2006), and providing a protective barrier against toxic substances (Pentreath and Slamon, 2000; Calabrese, 2008). Thus, chronic exposure to environmental chemicals, or inflammatory molecules from vasculature, may potentially affect the function of astrocytes and gliotransmitter release (Kim et al., 2014; Orellana et al., 2014; Avendano et al., 2015).

In this review, we present the latest methods that enable scientists to decipher the molecular mechanisms of gliotransmitter secretion. In particular, we focus on the vesicular exocytosis of gliotransmitters from astrocytes using optical microscopic imaging. We further discuss how genetic alterations, acute injuries, and chronically toxic conditions (including exposure to stress *in utero*) could impair gliotransmission and consequently lead to neuronal and behavioral disorders.

## Molecular mechanisms underlying the release of gliotransmitters

There have been two major methodological breakthroughs that have allowed for profound understanding of astrocytic activities including gliotransmission: calcium imaging and advanced optical microscopy (Li et al., 2013). The initial discovery made by using chemical calcium indicators was that astrocytes exhibit increased intracellular calcium concentration ([Ca^2+^]_*i*_), which spreads to adjacent astrocytes. This phenomenon is called Ca^2+^ waves (Cornell-Bell et al., 1990; Charles et al., 1991; Rusakov et al., 2014). Genetically encoded calcium indicators have enabled more detailed analysis of astrocyte functions (Shigetomi et al., 2013).

Two-photon microscopy enabled scientists to observe fluorescence with superior penetration depth. Thus, studies on astrocytes have been expanded to experiments using brain slices and *in vivo* models (Nimmerjahn et al., 2004; Nishida and Okabe, 2007). Moreover, thanks to total internal reflection fluorescence microscopy, which can visualize fluorescent molecule behaviors beneath the plasma membrane, the interaction between [Ca^2+^]_*i*_ elevation and subsequent vesicular trafficking became precisely clarified (Bezzi et al., 2004; Shigetomi et al., 2012; Oya et al., 2013).

**Figure 2:**
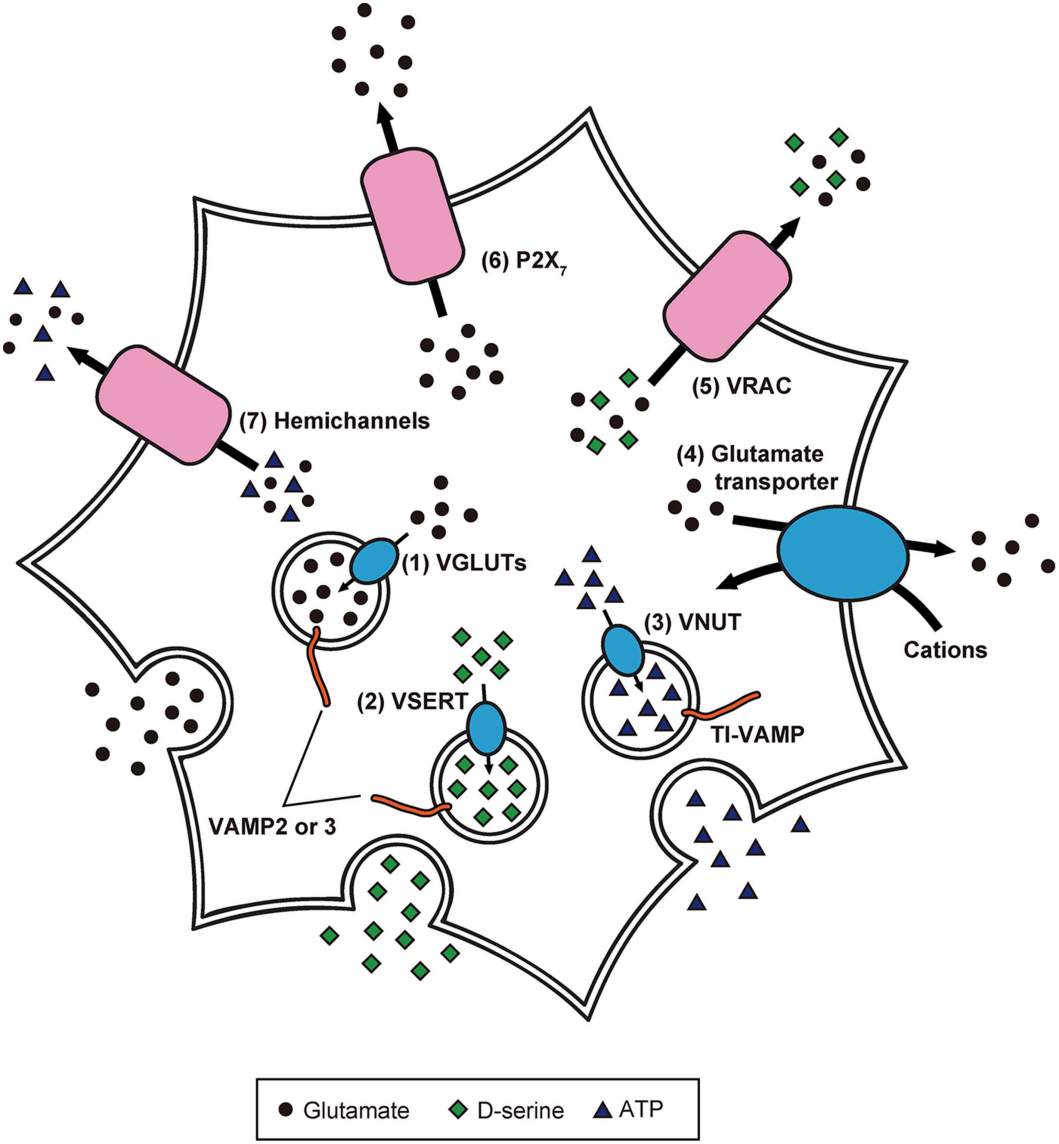
**Precise intracellular machinery involved in the release of glutamate, D-serine, and ATP from astrocytes**. Glutamate and D-serine are taken up into synaptic-like vesicles through (1) VGLUT and (2) vesicular D-serine transporters (VSERT), respectively. These synaptic-like vesicles fuse to the plasma membrane, mediated by SNARE proteins including VAMP2 or VAMP3, in response to [Ca^2+^]_*i*_ increase. In contrast, ATP is released through secretory lysosomes. Storage of ATP into secretory lysosomes is achieved by (3) VNUT. Through the interaction of SNARE proteins including TI-VAMP, ATP-containing secretory lysosomes are Ca^2+^-dependently exocytosed. Moreover, the existence of other release mechanisms has been discovered: (4) reverse operation of plasma membrane glutamate transporters, (5) cell swelling-induced anion transporter (VRAC) opening, (6) release via P2X_7_ receptors, and (7) gap junction channels (hemichannels) on the cell surface of astrocytes.

Because of these experimental advancements, accumulating evidence suggests the paradigm that: (1) inositol 1,4,5-trisphosphate-mediated Ca^2+^ release from endoplasmic reticulum causes [Ca^2+^]_*i*_ increases in astrocytes in response to the activity of adjacent astrocytes and neurons; (2) elicited [Ca^2+^]_*i*_ elevation induces release of gliotransmitters (Halassa et al., 2007; Oya et al., 2013; Khakh and McCarthy, 2015). Although the exact mechanisms of gliotransmission are unclear, recent studies have partially revealed the release mechanisms of glutamate, D-serine, and ATP in astrocytes (Figure 2; Gucek et al., 2012; Li et al., 2013).

## Glutamate

Although, glutamate is well-known as a neurotransmitter, it also acts as a gliotransmitter. Application of bradykinin to cultured astrocytes induces glutamate release and influences adjacent neurons through N-methyl-D-aspartate (NMDA) receptors (Parpura et al., 1994). In contrast, application of clostridium, tetanus, and botulinum neurotoxins, which differentially cleave the exocytosis-regulating soluble N-ethylmaleimide-sensitive factor attachment protein receptor (SNARE) proteins, reduces Ca^2+^-dependent glutamate release. These findings suggest that the SNARE proteins, including vesicle-associated membrane protein-2 (VAMP2), syntaxin-1, and synaptosome-associated protein-23, mediate Ca^2+^-dependent glutamate release (Montana et al., 2006; Parpura and Zorec, 2010).

The uptake of cytoplasmic glutamate into exocytotic vesicles is mediated by vesicular glutamate transporters (VGLUTs), which are driven by a proton gradient produced by vacuolar-type H^+^ ATPases (V-ATPases; Takamori et al., 2000; Gucek et al., 2012). Inhibition of V-ATPases blocks Ca^2+^-dependent glutamate release (Parpura and Zorec, 2010). Furthermore, VGLUT1 and 2 are colocalized with synaptic-like vesicles (Bezzi et al., 2004), suggesting that glutamate is packaged into synaptic-like vesicles and released from astrocytes in a Ca^2+^-dependent manner.

Meanwhile, other release mechanisms have been identified: (1) reverse operation of plasma membrane glutamate transporters (Longuemare and Swanson, 1995); (2) cell swelling-induced anion transporter opening (Kimelberg et al., 1990); (3) release via P2X_7_ receptors (Duan et al., 2003); (4) gap junction channels (i.e., hemichannels) on the cell surface of astrocytes (Ye et al., 2003). However, it is not clear how often and to what extent astrocytes employ these different mechanisms. Further studies will be needed to clarify whether there are specific release mechanisms that operate under particular conditions.

## D-serine

The discovery of D-serine as a gliotransmitter was remarkable because it was long thought that mammalian tissues only produced L-isomers of amino acids (Oliet and Mothet, 2006; Henneberger et al., 2012). D-serine is thought to be produced from L-serine by serine racemase (de Miranda et al., 2002). In cultured astrocytes, application of glutamate enhanced Ca^2+^-dependent secretion of D-serine via the activation of α-amino-3-hydroxy-5-methyl-4-isoxazolepropionic acid (AMPA)/kainate receptors (AMPA/KARs) and metabotropic glutamate receptors (Mothet et al., 2005). Correspondingly, agonists for AMPA/KARs and metabotropic glutamate receptors were found to increase [Ca^2+^]_*i*_ as well as subsequent secretion of D-serine, which is reduced by inhibition of these receptors. Furthermore, tetanus neurotoxins and V-ATPase inhibitors suppress agonist-evoked secretion of D-serine, and VAMP2/3 and VGLUT2-containing vesicles that are colocalized with D-serine. These results suggest that D-serine is stored in the synaptic-like vesicles and released from the vesicles in a Ca^2+^-dependent manner (Martineau et al., 2013).

## ATP

Although ATP is the primary energy currency of the cells, ATP can also act as a signaling molecule through purinergic receptors. A recent study showed that culture medium from cultured astrocytes exhibiting Ca^2+^ waves contained more ATP than control culture medium. Interestingly, the addition of collected culture medium to astrocytes induced Ca^2+^ waves that were inhibited by purinergic receptor antagonists (Guthrie et al., 1999). Thus, the ATP released from astrocytes induces Ca^2+^ waves, which astrocytes use to communicate with each other. However, the ATP release mechanisms still remain controversial; several lines of investigation have suggested various putative models for ATP release from astrocytes (Koizumi, 2010).

Connexin 43 (Cx43) assembles into a hemichannel which constitutes gap junctions in astrocytes, and exchanges signaling molecules, including Ca^2+^ and inositol 1,4,5-trisphosphate, between adjacent astrocytes (Orellana and Stehberg, 2014). Bioluminescence imaging of ATP combined with single channel recording showed that Cx43 hemichannels in rat glioma C6 cells and CA1 hippocampal astrocytes are permeable to ATP (Kang et al., 2008). Consistent with this finding, glutamate evoked [Ca^2+^]_*i*_ increase and ATP release in astrocytes of hippocampal slices, which were inhibited by application of a hemichannel blocker and in Cx43/Cx30 knockout mice (Torres et al., 2012), suggesting that ATP is released extracellularly through Cx43 hemichannels.

However, some studies have shown the involvement of secretory lysosomes in ATP release from astrocytes. In fact, primary cultured astrocytes express a secretory lysosome marker called vesicle-associated membrane protein-7 (also called TI-VAMP), and TI-VAMP-positive secretory lysosomes contain ATP which is Ca^2+^-dependently released (Verderio et al., 2012). In an experiment using primary cultured astrocytes and C6 cells, vesicular nucleotide transporter (VNUT)-positive lysosomes were labeled with fluorescent ATP, and application of VNUT inhibitor reduced the number of fluorescent ATP-containing vesicles. Observation by total internal reflection fluorescence microscopy revealed exocytotic events of secretory lysosomes in the cells following the application of a calcium ionophore, ATP, and glutamate. Thus, ATP is stored in lysosomes and released from lysosomes in a Ca^2+^-dependent manner (Oya et al., 2013).

## Contribution of gliotransmitter release to development and disease

Release of gliotransmitters regulates synaptic transmission between neurons and the extracellular environment in the brain. It is known that glutamate and D-serine excite synaptic transmission. However, whether ATP potentiates or inhibits synaptic transmission is still under debate because adenosine, a metabolite synthesized from ATP, usually inhibits synaptic activity via adenosine A_1_ receptors (Koizumi, 2010; Nam et al., 2012; Delekate et al., 2014). It is therefore reasonable to speculate that imbalance in the release of these gliotransmitters could result in altered neuronal activity. Various pathological conditions, including CNS diseases, traumatic brain injuries, developmental disorders, and prenatal exposure to deleterious molecules have been reported to be closely associated with impairment of gliotransmission.

## CNS diseases

Many CNS diseases are attributed to hyperactivity of neurons or unregulated neuronal cell death. Although such conditions have long been the focus of “neurocentric” studies, recent progress in the study of astrocytic gliotransmission has provided accumulating evidence for the contribution of astrocytes (Rossi et al., 2011; Verkhratsky et al., 2014).

Epilepsy is one of the most common CNS diseases, and is characterized by sudden and frequent seizures resulting from excessive firings by neurons (Wetherington et al., 2008). In slices from epilepsy model mice, astrocytic glutamate release was found to cause abnormal and prolonged depolarization in neurons (Tian et al., 2005). Furthermore, tumor necrosis factor-α (TNFα) and prostaglandins (PGs), released from astrocytes under traumatic events, can reactivate their calcium signaling, and can cause increased glutamate release (Bezzi et al., 1998, 2001; Domercq et al., 2006).

Reactive astrocytes are also involved in the pathogenesis of other neuronal disorders. In a mouse model of Huntington's disease, cultured astrocytes exhibited hyperactivated Ca^2+^-dependent glutamate release. This activity was owing to increased expression of pyruvate carboxylase (Lee et al., 2013), or reduced expression of glutamate transporter-1 and K_ir_4.1 K^+^ channels, which are key regulators for the clearance of extracellular glutamate and maintenance of membrane potentials, respectively (Behrens et al., 2002; Tong et al., 2014). In addition to overpotentiating neuronal activity, excessive accumulation of extracellular glutamate causes cytotoxicity. For instance, mice with genetic deletion in glutamate transporter-1 exhibit reduced glutamate clearance, and consequently display abnormal cell death in motor neurons, reminiscent of amyotrophic lateral sclerosis (Staats and Van Den Bosch, 2009).

In Alzheimer's disease (AD) mouse models, reactive astrocytes are detected near β-amyloid plaques (Nagele et al., 2004). Although chronic rise in [Ca^2+^]_*i*_ is a well-known phenomenon in reactive astrocytes in AD, its underlying mechanisms remain unclear. A recent study demonstrated that purinergic signaling through Cx43 hemichannels and P2Y1 receptors mediated the hyperactivity of astrocytes in AD (Delekate et al., 2014). Consistent with this finding, upregulation of Cx43 hemichannels was observed in an AD mouse model (Mei et al., 2010), and AD patients displayed higher levels of ATP in brain regions surrounding β-amyloid plaques (Mecheri et al., 1997; Mandal et al., 2012).

Gliotransmitter release from astrocytes is also required for correct development of neuronal circuits. In particular, glial-neuronal communication through NMDA receptors is an essential process for proper dendritic morphogenesis and establishment of synaptic connections (Rabacchi et al., 1992; Sin et al., 2002; Espinosa et al., 2009). Although NMDA receptors are activated by both glutamate and D-serine, recent discoveries suggest that D-serine plays an important role in dendritic development and long-term potentiation (Henneberger et al., 2010; Devito et al., 2011; Balu and Coyle, 2012; Diniz et al., 2012). Mice with a deletion of serine racemase showed reduced levels of brain D-serine and brain-derived neurotrophic factor, and loss of glutamatergic neurotransmission, and consequently had less complex dendrites (Morita et al., 2007; Balu and Coyle, 2012). Because NMDA receptor malfunction has been considered to be responsible for schizophrenia, deficiency in D-serine secretion from astrocytes can be a potent schizophrenia risk factor (Van Horn et al., 2013). Indeed, association studies of schizophrenia patients revealed several mutations in genes for serine racemase, as well as D-amino acid oxidase and its interacting protein G72 (Boks et al., 2007; Morita et al., 2007; Müller et al., 2011; Caldinelli et al., 2013).

## Injury and infection

Acute brain insults, caused by ischemia or infection, affect neuronal circuitry through direct inflammatory responses in neurons and through signals from glial cells (Vesce et al., 2007; Calì et al., 2009). Astrocytes under acute inflammatory conditions undergo reactive astrogliosis similarly to those in CNS diseases, albeit with differences in gene expression and cell structure (Khakh and Sofroniew, 2015). Upon injury or ischemia, damaged neurons, endothelial cells and glial cells are known to release considerable amounts of ATP (Cook and McCleskey, 2002; Wang et al., 2004; Davalos et al., 2005; Nedergaard et al., 2010). Increased levels of extracellular ATP activate purinergic receptors on astrocytes, particularly P2Y1 (Domercq et al., 2006), thereby inducing [Ca^2+^]_*i*_ elevation and release of glutamate, as well as ATP (Domercq et al., 2006; Nedergaard et al., 2010). Furthermore, inflammatory molecules including TNFα, interleukin-1β, and PGs, are profoundly engaged in these responses. Not only the activated microglia converge to the site of injury and secrete cytokines; astrocytes themselves synthesize TNFα and PGs (Domercq et al., 2006; Santello et al., 2011). TNFα and PGs either interact with certain processes in the stimulus–secretion coupling machinery within astrocytes (Domercq et al., 2006; Santello et al., 2011), or bind to TNFα and PGs receptors on astrocytes after secretion (Bezzi et al., 2001; Vesce et al., 2007).

## Chronic and prenatal exposure to chemicals

Increasing evidence shows significant correlations between environmentally deleterious chemicals and the risk of neurodevelopmental disorders (Feng et al., 1990; Leonardsson and Ny, 1997). Previous studies have focused on the effects of toxic substances on neurons, but recently it was suggested that astrocytes are also involved in the pathogenesis of those conditions.

Owing to their close connections with microvascular units via BBB, astrocytes tend to be chronically exposed to noxious molecules in circulation. Probably because of their interactions with environmental toxins, astrocytes possess more resilient and adaptive machinery against toxic molecules compared with neurons (Pentreath and Slamon, 2000; Calabrese, 2008). These protective systems include the glutathione system, superoxide dismutase, and hemeoxygenase (Dwyer et al., 1995; Huang and Philbert, 1995; Blaauwgeers et al., 1996; Pentreath and Slamon, 2000). Nevertheless, excessive passage of harmful substances across the BBB seriously affects astrocyte homeostasis and functionality.

The toxicological effects of heavy metals (e.g., mercury, zinc, manganese, and aluminum) on neurons and glial cells have been studied for decades (Calabrese, 2008; De Keyser et al., 2008). However, it is unclear how these metals affect gliotransmitter release. Some studies have shown that lead and manganese induce cytotoxic cell death by impairing glutamate uptake in astrocytes (Normandin and Hazell, 2002; Struzynska et al., 2005). However, pathological effects on gliotransmission by lifestyle-associated factors, such as smoking, drinking, and insufficient sleep, are becoming the focus of growing interest. Because nicotinic acetylcholine receptors are expressed on astrocytes, they exhibit nicotine sensitivity and [Ca^2+^]_*i*_ increase (Oikawa et al., 2005; Delbro et al., 2009). Ethanol causes reactive oxygen species production, and [Ca^2+^]_*i*_ increase and glutamate secretion from astrocytes (Salazar et al., 2008). Astrocytes exposed to ethanol also exhibit alterations in Golgi complex morphology, secretory vesicle biogenesis, and expression levels of Rab GTPases and motor proteins (Tomas et al., 2005), which may be an additional factor for the dysfunction of brain development caused by ethanol.

Because adenosine plays a critical role in the control of sleep-wakefulness (Thakkar et al., 2003), and chronic alcoholism is frequently accompanied by sleep disorders (Brower, 2001), changes in sleep pattern may also induce alteration in gliotransmitter release. Interestingly, hypothalamic astrocytes from rats following sleep deprivation exhibited different proteome profiles, and the expression of VAMP2, which is an essential protein for vesicular exocytosis (Kim et al., 2014), was significantly increased. These findings suggest a strong association between alcohol intake, sleep disorders, and astrocytic gliotransmission.

Additionally, certain ambient ultrafine particles, which are defined as particulate substances with a diameter less than 100 nm, are emerging as another toxic substance that may deleteriously affect brain function (Block and Calderón-Garcidueñas, 2009; Loane et al., 2013). In a recent study, ultrafine carbon black, a surrogate for ultrafine particles, was shown to induce the release of glutamate and ATP from astrocytes by activating Cx43 and pannexin-1 hemichannels (Wei et al., 2014).

Recent epidemiological and experimental studies have demonstrated that children born from mothers who are exposed to infections or are addicted to alcohol or drugs have a higher risk of neuronal disorders and abnormal behaviors (Jacobsen et al., 2006; Stringari et al., 2008; Boksa, 2010; Brolese et al., 2015). However, the effects of these agents on astrocytes still remain largely unknown. Some studies have shown that prenatal exposure to lipopolysaccharides or nicotine together with postnatal high-fat/cholesterol diet result in enhancement of Cx43 hemichannel activity, and consequently increases the release of glutamate and ATP (Orellana et al., 2014; Avendano et al., 2015).

## Conclusions

Over several decades, researchers have attempted to understand the properties and pathologies of the CNS by focusing solely on neurons; however, recent improvements in molecular and cellular imaging techniques are increasingly indicating that this neurocentric approach needs to be revised. In addition to neurons, glial cells including astrocytes are important elements for brain functions. Astrocytes are located in close morphological and functional relationships with blood vessels and neurons, and various genetic or environmental factors are implicated in gliotransmission impairment. Considering these characteristics of astrocytes, further studies will provide new insight on the significance of gliotransmitter release for fetal neurodevelopment. Thus, new therapies can be developed to overcome environmental chemical-induced neurodevelopmental disorders.

## Author contributions

KH, TK, and TT wrote the paper.

## Funding

This work was funded in part by a research grant from The Grant of National Center for Child Health and Development, Tokyo, Japan (grant numbers 27-9), and by a Grant-in-Aid for Science Research from the Ministry of Education, Culture, Sports, Science, and Technology of Japan.

### Conflict of interest statement

The authors declare that the research was conducted in the absence of any commercial or financial relationships that could be construed as a potential conflict of interest.
